# Rhythm is it: effects of dynamic body feedback on affect and attitudes

**DOI:** 10.3389/fpsyg.2014.00537

**Published:** 2014-06-10

**Authors:** Sabine C. Koch

**Affiliations:** ^1^SRH University HeidelbergHeidelberg, Germany; ^2^University of HeidelbergHeidelberg, Germany

**Keywords:** embodiment, body feedback, approach and avoidance motor behavior, attitudes, movement qualities, movement rhythms, movement analysis, dance movement therapy

## Abstract

Body feedback is the proprioceptive feedback that denominates the afferent information from position and movement of the body to the central nervous system. It is crucial in experiencing emotions, in forming attitudes and in regulating emotions and behavior. This paper investigates effects of dynamic body feedback on affect and attitudes, focusing on the impact of movement rhythms with smooth vs. sharp reversals as one basic category of movement qualities. It relates those qualities to already explored effects of approach vs. avoidance motor behavior as one basic category of movement shape. Studies 1 and 2 tested the effects of one of two basic movement qualities (smooth vs. sharp rhythms) on affect and cognition. The third study tested those movement qualities in combination with movement shape (approach vs. avoidance motor behavior) and the effects of those combinations on affect and attitudes toward initially valence-free stimuli. Results suggest that movement rhythms influence affect (studies 1 and 2), and attitudes (study 3), and moderate the impact of approach and avoidance motor behavior on attitudes (study 3). Extending static body feedback research with a dynamic account, findings indicate that movement qualities – next to movement shape – play an important role, when movement of the lived body is an independent variable.

## INTRODUCTION

Movement is central to the human condition (Sheets-Johnstone, 1999). It is dynamic and as pervasive as the air that we breathe. Movement therapies use this basic human capacity in order to restore health, access resources, and diminish suffering (e.g., Koch et al., 2013). Movement also provides us with central cues for indications and can predict therapy outcomes (e.g., Ramsayer and Tschacher, 2011). Dance movement therapy (e.g., Levy, 2005) assumes that next to the *shape* of a movement (e.g., approach vs. avoidance movement), its’ *quality* is of central importance (Kestenberg, 1995; Laban, 1960). Next to the *what*, the *how* of the movement (e.g., indulgent vs. fighting movement) can have an influence on our affect and attitudes, cognition and interpersonal relations (Kestenberg and Sossin, 1973; Stern, 1985; Rizzolatti, 2013).

With the change from a computer metaphor-based to a more organismic understanding of the human condition (Smith and Semin, 2004) in embodiment research, human movement has moved back into a scientific focus. Research in psychology and neuroscience demonstrated that the observation of the movement of our conspecifics in a goal related task sets of our own motor programs in order to understand the intentions of the others (e.g., Buccino et al., 2001). The bodily reactions of our conspecifics cause empathic bodily reactions in ourselves (Bavelas et al., 1986; Chartrand and Bargh, 1999; Wilson and Knoblich, 2005); and the congruency of motor behavior with a cognitive task influences the effectiveness of our performance (e.g., Wells and Petty, 1980; Förster and Strack, 1996).

Body feedback from postures can cause differential affect (Strack et al., 1988), attitudes (Cacioppo et al., 1993; Neumann and Strack, 2000; Maass and Russo, 2003; Förster, 2004; Schubert, 2004), and cognition (Mussweiler, 2006; Cuddy et al., 2012; for a review of these effects see Niedenthal et al., 2005). Since most of the latter studies have focused on static but not dynamic body feedback, it seems timely to analyze influences of movement on affect, attitudes, and cognition, and to specify clinical implications of these findings.

The expressive function of movement has been a scientific topic ever since Darwin had published “The expression of emotions in men and animals” (Darwin, 1872/1965). The impressive function of movement (Wallbott, 1990) has been a focus in psychology starting with James–Lange theory, and has thrived empirically with the postulation of the facial feedback hypothesis (Buck, 1980; Laird, 1984). Body feedback approaches have subsequently further extended to include postural feedback (e.g., Riskind, 1984; LaFrance, 1985; Rossberg-Gempton and Poole, 1992), and vocal feedback (Hatfield et al., 1995; for a general overview on body feedback research see Hatfield et al., 1994) but most of this research has remained in the static realm of held postures or facial expressions to date.

For clinical applied fields such as body psychotherapy or dance movement therapy working with movement of the lived body as an independent variable all the time, body feedback research needs to move on to investigate effects of movement on affect, cognition, and health-related outcomes. The dynamic character of movement has not yet been fully accounted for by embodiment research in general and body feedback research in particular, which so far focused on effects of static facial expressions and postures on affect and cognitions. Movement, however, is characterized and defined by its dynamic properties: its relation to space, weight (gravity/force), and time (Laban, 1960), and its proprioceptive and kinesthetic properties (Sheets-Johnstone, 1999; Gibbs, 2006). In body feedback studies using held postures, these properties of movements have not been taken into account leading to a lack of knowledge if it comes to effects of movement interventions. Moreover, almost all embodiment research so far has focused exclusively on movement shape (i.e., changes in the form or direction of the movement), and has not considered changes in movement quality (i.e., changes in muscle tension and the dynamic properties related to space, weight, and time).

### ROUND vs. SHARP REVERSALS

An exception is the study of Aronoff et al. (1992) who considered the impact of movement qualities on perception. Aronoff et al. (1992) investigated emotional implications of round vs. angular movement (study 1), and round and angular facial cues (study 2) reporting that round properties are related to perception of emotional warmth, cordiality and positive roles of actors on stage, whereas angular properties are related to perceptions of threat and negative roles of actors on stage. Angular shapes and sharp transitions had already been demonstrated to cause more attributions of aggressiveness in the classic movie of Heider and Simmel (1944) on the antropomorphization of animated geometric forms. In a similar vein, a study of Bar and Neta (2006) using stimuli from everyday objects (watches, sofas, etc.) found that attitudes toward curved shapes were significantly more positive than attitudes toward sharp-angled shapes. This basic smooth vs. sharp distinction was already found in a classic experiment by gestalt psychologist Köhler (1929). People were asked to assign the names *bouba* and *kiki* to one of two shapes (later also exchanged by the words *maluma* and *takete*), one looking like a round-curved inkblot and the other like a sharp-edged star. Between 95 and 98% of people asked assigned *kiki* to the angular shape and *bouba* to the rounded shape. The effect has been demonstrated in different cultural contexts and also in children as early as two and a half years of age (Maurer et al., 2006). Yet, Aronoff et al. (1992) remain the only researchers to have empirically looked at these properties in movement (study 1). However, they focused on perceptual effects and did not account for body feedback effects (i.e., the effects from peripheral movement on more central processes such as affect or cognition), nor did they explicitly distinguish movement shape from movement quality. Our findings extend Aronoff’s work with a body feedback approach, distinguishing the effects of movement shape from the effects of movement qualities more explicitly than Aronoff et al. (1992).

### A THEORY ON MOVEMENT AND MEANING

Early attempts to specify dynamic movement qualities have been made by movement analysts^1^ (e.g., Laban, 1960) and later have been selectively related to psychological properties (e.g., Kestenberg, 1995). One of the most complete and differentiated theory-systems on how movement maps to semantics is the Kestenberg Movement Profile (KMP; Kestenberg and Sossin, 1973, 1979; Kestenberg-Amighi et al., 1999; Koch and Sossin, 2013). With a focus on clinical and developmental applications such as early mother–child interaction, Kestenberg developed nine perspectives on movement (yielding nine diagrams) based on the three dimensions of space, weight (gravity/force) and time, and the three planes of horizontal, vertical and sagittal movement. Following Laban (1960) and Lamb (1965), Kestenberg distinguished two basic movement systems: movement qualities and movement shape. On the basis of psychodynamic theories (Freud, 1965), she related those to the first years of child development as well as to clinical issues and personality traits in the adult. She thereby offered a comprehensive theory-system for a wide range of applications in non-verbal diagnosis and intervention. Since her predictions are directly related to human movement as an observable independent variable her theory is testable and offers a wealth of hypotheses to clinical embodiment research. This article focuses on the movement rhythms and the underlying principles of the Kestenberg system (for a more complete account on the KMP see Kestenberg-Amighi et al., 1999; Koch and Sossin, 2013).

### MOVEMENT RHYTHM

**FIGURE 1:**
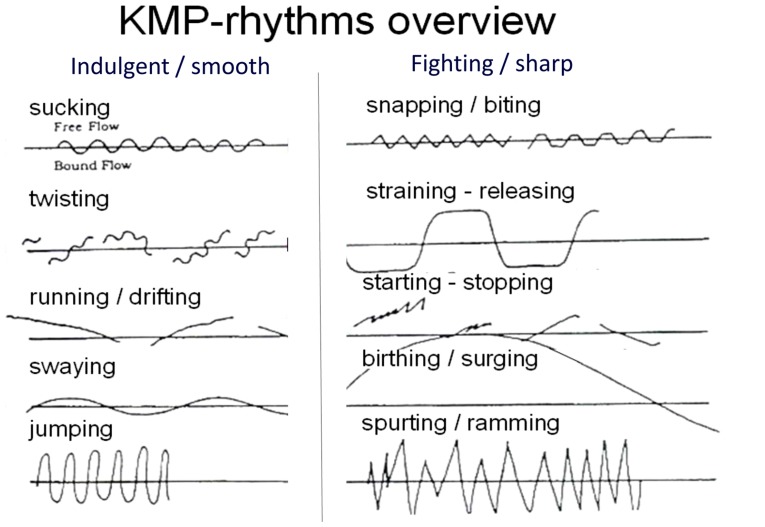
**Overview of the Kestenberg tension-flow rhythms.** The 10 prototypical developmental movement rhythms following KMP-theory (cf. Kestenberg-Amighi et al., 1999). Study 1 used jumping vs. spurting/ramming rhythm, study 2 used swaying vs. snapping/biting and starting–stopping rhythm, and study 3 used sucking and jumping vs. starting–stopping and spurting/ramming rhythm (depending on the intensity and timing participants used). Rhythms may vary in three sets of parameters (*attributes*): regularity of amplitude (*even* vs. *flexibile*), intensity-level (*high* vs. *low intensity;* indicated in height of vertical stroke), and timing (*abrupt* vs. *gradual;* indicated in steepness of slope), and are related to the three dimensions of space, weight and time respectively while rhythms indicate needs on the individual level, shapes indicate relations to persons or objects.

When we hear of rhythms we may think of music rather than of movement. Yet, just like there are external rhythms that can make us move to the beat (Grahn and Brett, 2007), there are internal ones that are related to our own situational needs and affect. They are expressed by the constant subtle alternations in muscle tension and relaxation in the body (Kestenberg and Sossin, 1973, 1979; Kestenberg, 1995). KMP-theory distinguishes 10 prototypical movement rhythms^2^ (see **Figure 1**) that correspond to physiological and psychological needs of a person (Kestenberg, 1995; Kestenberg-Amighi et al., 1999). They belong to the broader system of movement qualities and fall in two basic categories: indulgent rhythms and fighting rhythms. Indulgent rhythms have smooth reversals (reversals are the transitions from tension to relaxation and vice versa; examples for smooth rhythms are sucking or swaying) and serve joyful indulgence into new behavior, while fighting rhythms have sharp reversals (e.g., snapping transitions such as in cutting or biting) and serve necessary separation from old behavior and defense against outer or inner demands. These movement rhythms already start to develop in the fetal stage (Loman, 2007). In each developmental phase, one indulgent rhythm that facilitates acquisition, mobilization into new patterns, and libidinal repetition of predominant movements precedes a fighting rhythm that facilitates stabilization, differentiation, and separation from that particular phase (cf. Erikson, 1950). The sucking rhythm is the first rhythm that organizes the body of the child. It has smooth reversals, low intensity, and regular amplitudes spreading from the mouth to all other body parts, particularly when children need to soothe themselves, for example, immediately before falling asleep (Lotan and Yirmiya, 2002). The biting rhythm has sharp reversals and helps the child to cut and separate things, first with the teeth, then with the hands and the entire body. It later serves analytic thinking and separation of categories, and can be observed, for example, when we bite our pencils or finger nails. Thus finding oneself biting on a pen might indicate the need to focus, concentrate and get concepts straight; if one finds oneself curling one’s hair or rocking in a sucking rhythm this can indicate the need to soothe oneself. Likewise, these rhythms are employed to address needs of others (e.g., soothing a baby; consider that all lullabies consist of sucking rhythms) and usually lie at the implicit level of our experience.

The method of rhythms notation, done in hand-writing on a blank sheet with a time line, makes use of kinesthetic empathy (Kestenberg, 1995). Observing the target person, notators take the changes in muscle tension and relaxation into their own body (by motor simulation) and from there into their writing arm, fingers and pencil (as bodily extension), finally producing a “tension-flow line” on the sheet. By convention, moving the pencil down thereby indicates an increase of tension in the body of the target, while moving the pencil up indicates a decrease of tension in the body of the target. Once notated, the rhythms are categorized and counted. Rhythm counts usually yield inter-rater reliabilities of *Cronbach’s Alphas* between 0.74 and 0.91 (Sossin, 1987; Koch, 2006).

### MOVEMENT SHAPE

Cacioppo et al. (1993) conducted an experimental series on approach and avoidance motor behavior, demonstrating that the application of pressure toward the body from below a table (held approach movement) produced more positive attitudes toward Chinese ideographs (arbitrary characters) than the application of pressure away from the body from above the table. The experiment was groundbreaking in two respects: it showed that the movement of the basic categories *approach and avoidance* directly produced pronounced attitudes, and researchers consciously applied the first dynamic variable, i.e., the application of directional force, to demonstrate the meaning of directional movement (toward and away from the body). Neumann and Strack (2000) replicated and extended these findings in Germany.

KMP-theory postulates that changes in shape-flow (i.e., rudimentary directional movement on the basic dimensions of growing/open vs. shrinking/closed) are related to affect and attitudes: approach behavior is expected to be related to positive affect and attitudes, avoidance behavior is expected to be related to negative affect and attitudes (e.g., the child grows toward the smiling mother; the child shrinks away from the angry dog). Shape-flow movements toward and away from the body (shape-flow design) are related to self-object-differentiation as well as to giving and taking. Cacioppo et al. (1993) use an evolutionary account to explain the effect of approach and avoidance motor behavior on attitudes: during ontogenesis – and also phylogenesis – persons have learned to take in good things (e.g., food) and to push away bad things (e.g., angry persons); this life-long learning process causes a conditioned evaluative preparedness of our cognitive-affective system. Similarly, Eberhard-Kaechele (2007) points out that in KMP-theory shape-flow differentiates on a preconscious level between toxic and nourishing stimuli and provides the appropriate response (i.e., growing toward or shrinking away from a stimulus). Thus, KMP-theory predicts an effect of movement qualities on affect and of movement shape on affect as well as on attitude.

### HYPOTHESES

Based on the approaches of Kestenberg and Sossin (1979) and Cacioppo et al. (1993), and on the grounds of theories of embodied cognition (Barsalou, 1999; Niedenthal et al., 2005; Niedenthal, 2007), we predicted how movement will influence affect and attitudes. The study extends existing findings in body feedback research using dynamic movement instead of statically held postures. Following KMP-theory, movement quality was included through the manipulation of movement rhythms with smooth vs. sharp reversals assuming that smooth rhythms would cause more indulgent affect in movers.

We employed two one-factorial (smooth vs. sharp rhythms) and one two-factorial (movement rhythm × movement shape) designs. Studies 1 and 2 tested the hypotheses that indulgent vs. fighting movement rhythms (smooth vs. sharp rhythms) would cause congruent^3^ answers on cognitive and affect measures (one-factorial between-group design). Study 3 tested the hypotheses that approach vs. avoidance motor behavior and smooth vs. sharp movement rhythms would cause congruent answers on an affect and attitude measure. The relative magnitude of the main effects and the interaction was explored (2 × 2 between-group design).

## STUDY 1: DYNAMIC BODY FEEDBACK FROM MOVEMENT RHYTHMS ON AFFECT AND COGNITION

Study 1 focused on systematic effects of dynamic body feedback from rhythms on affect and cognition. In addition to the expectation that movement rhythms with smooth reversals would cause more positive affect, we expected two motor congruency effects on the cognitive level. In a categorization task (online embodiment; i.e., embodiment effects directly caused in the situation), participants in the indulgent groups were expected to categorize “smooth” words (e.g., *sway*) faster than “sharp” words (e.g., *bite*), and participants in the fighting groups were expected to categorize “sharp” words faster than “smooth” words. In the memory task of study 1 (offline embodiment; i.e., embodiment effects from memory), participants were expected to remember more congruent words, respectively.

## METHOD

### SAMPLE

Sixty participants (30 women, 30 men; mean age = 23.83; SD = 8.54) were tested in a one-factorial between group designs. Thirty used jumping rhythm (indulgent; smooth reversals), and thirty used spurting/ramming rhythm (fighting; sharp reversals). Participants had been either recruited in the psychology department at the local university or in the local central pedestrian zone (about 50% from each location). Most participants were students. In all studies, we matched men and women to the otherwise randomized groups. Gender was controlled in all studies but did not account for any differences related to the main hypotheses. Participants signed an informed consent form before the experiments started. A debriefing was provided at the end of the experiments. In all studies, participants received either course credit or sweets for their participation.

### COVER STORY

Participants were told in the beginning that this experiment aimed to measure the influence of different levels of physical arousal on a number of tasks. In all three studies, their pulse was taken before and after the movement, and served as a control variable, but did not have any significant influence. Their attention was thus turned away from the movement qualities.

### MOVEMENT MANIPULATION

We chose jumping rhythm vs. spurting/ramming rhythm as examples of indulgent vs. fighting rhythms because due to their high intensity and magnitude they were particularly easy to observe and embody, and particularly clear and easy to distinguish from one another (**Figure 1**). Participants in the indulgent condition were told to bounce on both feet, almost as if rope skipping, but without leaving the floor; those in the fighting condition were told to kick an imaginary ball with the left and right leg in alternation. Both movements were performed in high intensity and abrupt, differing merely in smoothness vs. sharpness of reversals (bouncing/jumping: smooth; kicking: sharp). Movements were performed for ~2 min, while participants categorized verbs into *smooth* (“*rund”)* and *sharp (“eckig”)* by mouse-clicks.

### INSTRUMENTS AND SCALES

#### Reaction time measure

Reaction times were measured for the semantic categorization task. Participants had to categorize 22 pretested verbs into the two categories of “smooth” or “sharp.” Verbs were taken directly from the rhythms terminology of the KMP translated to German, for example, “swaying” (wiegen) or “sucking” (saugen) for *smooth*, and “biting” (beissen) or “knocking” (klopfen) for *sharp*. We conducted a pretest with 20 participants and finally only used words correctly categorized by at least 14 persons. The presentation was programmed in Experimental Runtime System (ERTS, Beringer, BeriSoft, Frankfurt, Germany), verbs were presented in random order. Reaction time was measured computing the duration from display of the verbs to the mouse click by the participant.

#### Recall

The recall of the formerly categorized words was to be given in free format. We calculated with both number of “smooth” and “sharp” words recalled.

#### Affect measure

**FIGURE 2:**
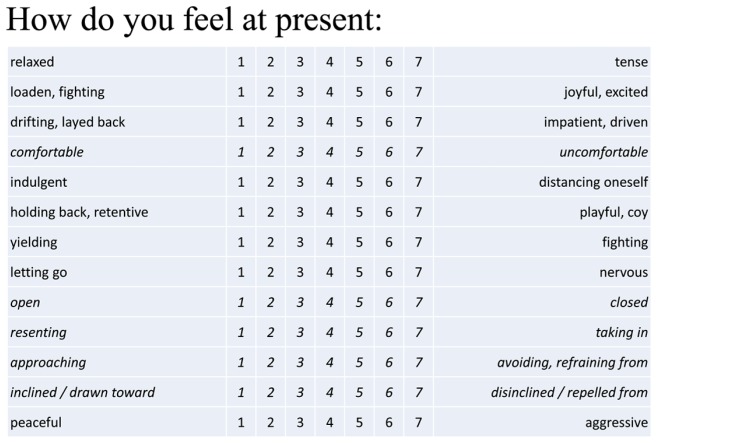
**Movement-based affect scale (MBAS; Koch and Müller, 2007).** The movement-based affect scale (13 items; originally “Brief KMP affect scale,” Koch and Müller, 2007) consists of eight items related to movement qualities and five items related to movement shapes and is based on the tension-flow and shape-flow concepts of Kestenberg (1995), Kestenberg and Sossin (1973, 1979); items related to movement shape are set in italics; those were added for study 3, when movement shape was introduced. All items have been derived from the KMP textbook of Kestenberg-Amighi et al. (1999) via the KMP-questionnaire (Koch and Müller, 2007) yielding good internal consistencies of scales of the German version (with *Cronbach’s Alphas* between 0.70 and 0.95). The affect scale in our studies showed *Cronbach’s Alphas* of 0.70 and 0.82 (without shape items in studies 1 and 2) and of 0.89 including shape items (in study 3).

Participants were asked “How do you feel? Please take some time to sense the effects of the movement just performed.” We employed a self-constructed movement based-affect scale (MBAS; Koch and Müller, 2007; **Figure 2**; non-italicized items) consisting of seven bipolar adjective items on a 7-point scale from a longer pretested version of the affect scale (KMP-questionnaire; Koch and Müller, 2007) containing the interpretative semantic terms from KMP-theory from the KMP-book by Kestenberg-Amighi et al. (1999) on the level of movement rhythms (indulgent vs. fighting; Koch and Müller, 2007). Sample items were *tense* vs. *relaxed, nervous* vs. *letting go*, etc., *Cronbach’s Alpha* was 0.70. Factor analysis revealed the expected one-factor solution with 63% of the variance explained. We used the sum score for computations. After reversion of polarization, higher values indicated more negative affect.

### PROCEDURE

Informed consent was obtained for participation and the fact that the session was video-taped. Then participants received the following instruction: “In this study we investigate the effects of bodily exhaustion on performance in a number of areas. For each of these areas you will complete a short task.” Heart rate and blood pressure were then obtained as a base-rate. Participants received instructions on how to move in the according condition (jumping vs. spurting ramming rhythm) with a short description and demonstration by the experimenter. On correct repetition, the person was asked to rehearse the movement for 15 more seconds before moving on to the first task. Subsequently, they received instructions for the categorization task. They had to hold a wireless mouse in both hands and categorize verbs that appeared via beamer on a white 2 m × 2 m screen in a distance of about 2 m. Words had to be categorized as fast as possible into the categories smooth vs. sharp by pressing the right or left mouse button using the right or left thumb respectively. After two exercise trials the categorization task started. Participants had to continue to perform the movement during the entire categorization task (~2 min). Immediately after the task, heart rate and blood pressure were taken again. Then the experimenter asked the participants to do the movement for another 15 s focusing on “how the movement feels” (without being distracted by the categorization task). Thereafter, participants had to characterize their impression using the affect scale (**Figure 2**). After that, participants had to recall as many words as possible from the categorization task writing them on a blank sheet. Finally, participants completed a demographic data sheet and were then debriefed about the aims of the study. In the end, they either received course credit or selected a small present from a selection of sweets. The study took ~30 min altogether.

### DATA ANALYSIS

We computed an analysis of variance (ANOVA) with movement rhythm (jumping vs. spurting/ramming) as independent variable, and reaction times, recall, and affect (measured with the MBAS) as dependent variables, using SPSS (2002, SPSS Inc., Chicago, IL, USA) and an 0.05 alpha-level.

## RESULTS AND DISCUSSION

Results indicate that indulgent vs. fighting rhythms led neither to a faster classification of congruent words, nor to a more frequent recall of congruent words (cognitive measures). They did, however, cause congruent affect in participants *F*(60,1) = 4.34, *p* = 0.042, η^2^ = 0.07. Descriptive statistics are provided in **Table 1**. The use of indulgent vs. fighting rhythms affected the affective level in the hypothesized direction: participants who performed indulgent movement felt more relaxed, joyful, indulgent, peaceful, playful, etc. Whereas participants who performed fighting movement felt more tense, intruding, fighting, aggressive, retaining, etc. The “missing effect” on the cognitive measures may indicate that movement qualities – at least on the rhythm level, i.e., the earliest and most implicit level – do not affect cognition, but it could also mean that our cognitive measures were not sensitive enough to the experimental manipulation or on a different cognitive level. We speculated that – consistent with KMP-theory – an evaluative measure may have been more adequate than a reaction time measure.

**Table 1:** Descriptives of studies 1 and 2.

	*Study 1 (N = 60)*	
	*Smooth*	*Sharp*
	*M (SD)*	*M (SD)*
Reaction times (in ms)	1780 (337)	1731 (298)
Recall (M freq words)	2.02 (1.35)	1.40 (0.96)
Affect (sum)*	23.23 (5.56)	26.43 (6.12)
	***Study 2 (N = 62)***	
	***Smooth***	***Sharp***
	***M (SD)***	***M (SD)***

Face evaluation^a^	-6.62 (11.65)	-8.21 (15.35)
Face recognition^b^	4.75 (0.43)	4.60 (0.56)
Affect (sum)**	40.03 (11.19)	49.13 (14.40)

Affect (sum) = sum value of the affect scale; higher values indicate more negative affect; ^a^judgment of 60 faces on a scale from –100 (very unsympathetic) to +100 (very sympathetic); ^b^recognition of 10 previously seen faces among 40 faces (frequency); *p < 0.05, **p < 0.01.

In sum, the manipulation of prototypical movement rhythms as basic dimensions of movement qualities showed the hypothesized effects on the affective level only. In order to investigate whether the changes in affect were in fact due to differential effects of movement qualities (indulgent vs. fighting), or just to this particular combination of rhythms (jumping vs. spurting/ramming), to any laterality effects (alternating vs. parallel leg movement), or even just to the very specific movements used in study 1 (pretending to bounce similar to rope-jumping vs. pretending to kick a ball), we conducted study 2. One of the cognitive measures was replaced by an evaluative measure in order to further investigate which dependent variables are generally affected by movement rhythms.

## STUDY 2: CONSISTENCY OF DYNAMIC BODY FEEDBACK FROM MOVEMENT RHYTHMS

In order to investigate the generalizability of the effects of study 1 to other combinations of rhythms, we conducted a second study similar to the first study using swaying rhythm (indulgent/smooth) vs. biting rhythm (fighting/sharp). Variables, design, cover story, instruments, procedure and hypotheses were parallel to those of study 1, except for the replacement of the categorization and recall task by a face evaluation and recognition task.

## METHOD

### SAMPLE

Sixty-seven participants, mostly psychology students from the local university, were tested. Sixty-two (22 men, 40 women; mean age = 22.75; SD = 3.97) were included into the final analyses. The others did not perform the movement correctly or consistently enough, as determined by a blind expert rater. Students received course credits for participation.

### MOVEMENT MANIPULATION AND HYPOTHESES

Participants sat on a table and swung their legs alternatingly (using swaying rhythm), or had to pull up their feet in parallel and push them down again (flexion and extension of foot ankle using biting rhythm, sometimes starting–stopping rhythm; this variation, however, was not important as long as it was a fighting rhythm) while they performed the evaluation task on a laptop. During the performance of the movement they had to push and hold down either a right or a left key to indicate the degree of sympathy of 60 neutral-expression stimulus faces (from -100 very unsympathetic to +100 very sympathetic). In addition to the motor congruency effect on affect, that is, that the smooth rhythm would again cause more positive affect, we expected two cognitive motor congruency effects: (a) higher sympathy ratings in the smooth rhythms group and (b) an increased recognition of the known faces where movement had been congruent to the valence of the initial evaluation. In the recognition task, they received 40 neural-expression facial stimuli: 10 known and 30 unknown. The 10 known were the ones they formerly had rated most extreme: their five most sympathetic and their five most unsympathetic. We computed an ANOVA using a 0.05 alpha-level with movement rhythm (swaying vs. biting/starting–stopping) as independent variable, and evaluations of faces, recognition of faces, and affect (MBAS) as the dependent variables. *Cronbach’s Alpha* for the MBAS was 0.82.

## RESULTS AND DISCUSSION

**FIGURE 3:**
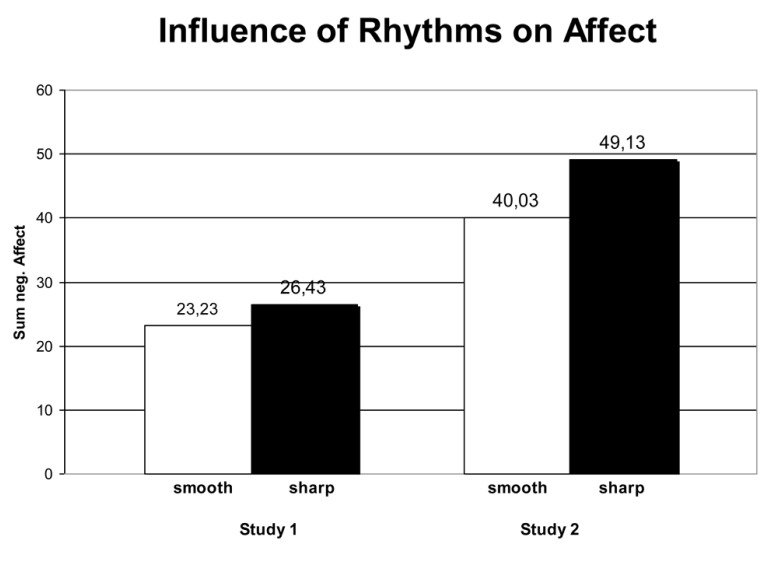
**Affect ratings (sum values) after indulgent vs. fighting movement in study 1 and 2.** Results of studies 1 and 2: fighting rhythms caused higher negative affect (*tense, aggressive, nervous,* etc.), whereas indulgent rhythms caused higher positive affect (*relaxed, joyful, playful,* etc.); higher values indicate more negative affect.

In line with our assumptions, findings were almost identical to study 1. We found no effects of indulgent vs. fighting rhythms on the cognitive-evaluative measure (neither online nor offline), but an effect on the affective measure *F*(1,62) = 7.77; *p =* 0.007; η^2^ = 0.12. Again, the use of indulgent vs. fighting rhythms particularly affected the affective level in the hypothesized direction: when participants performed indulgent movement they felt more relaxed, joyful, etc.; when they performed fighting movement they felt more tense, aggressive, etc. Study 2 thus replicated the results of the first study. In sum, studies 1 and 2 demonstrate the initial validity of indulgent (smooth) vs. fighting (sharp) movement qualities as a meaningful basic dimension of movement and their link to the affect system as hypothesized by KMP-theory (Kestenberg, 1995). Cognition, also in the more evaluative operationalization, remained unaffected (see **Table 1**; **Figure 3**). Since the gist of these findings corresponded to the predictions of KMP-theory, and at the same time added the essential movement qualities to body feedback research, we felt encouraged to carry our studies further. On the basis of our results, we speculated that while fighting vs. indulgent movement may not have caused a main effect on our first evaluative measure selected, it may still affect other evaluative measures, maybe in conjunction with other embodiment effects on attitudes.

## STUDY 3: DOES MOVEMENT QUALITY MODERATE APPROACH AND AVOIDANCE MOTOR EFFECTS?

In body feedback research, there is a well-known effect of arm flexion and extension causing more positive vs. more negative attitudes toward initially valence-free stimuli (Cacioppo et al., 1993). With a series of six experimental studies, Cacioppo et al. (1993) were among the first researchers to take the entire body plus a held directional force into account as an independent measure influencing attitude formation. They showed that non-facial and rudimentary dynamic motor manipulations can influence participants’ attitudes toward initially valence-free stimuli (Chinese ideographs). Participants either performed an approach movement (i.e., arm flexion: they pressed their palms against the underside of a table, thereby mobilizing force upward and toward the body) or an avoidance movement (i.e., arm extension: they pressed their palms against the surface of a table, thereby mobilizing force downward and away from the body). While performing the movement, participants watched a series of 24 initially valence-free Chinese ideographs. When they later evaluated the ideographs, participants in the approach condition rated the ideographs significantly more positively than participants in the avoidance condition. These findings have been supplemented by empirical studies identifying moderators such as laterality (Cretenet and Dru, 2004; Dru and Cretenet, 2005), hemispheric processing asymmetries, and personality traits (Maxwell and Davidson, 2007), as well as the valence of the stimuli, and the relation to the effects’ situated meaning (Centerbar and Clore, 2006). All of these studies have begun to take movement into account by inducing a basic movement direction, and by this means bringing rudiments of goal direction into the equation. Cacioppo et al. (1993) interpreted their findings as a direct effect of motor behavior on attitude. KMP-theory suggests that, because of their strong relation to affect, rhythms with smooth vs. sharp reversals should influence rudimentary attitudes in a similar fashion as approach vs. avoidance motor behavior; they may thus be components of, or contributors to, attitudes.

In this study, we aimed to replicate and extend the results from Cacioppo et al. (1993) that arm flexion and extension (as a manipulation of movement shape) has a differential effect on attitudes toward valence-free stimuli. Since we were interested in the effects of movement proper, rather than mere expense of held force, participants were instructed to move their arms rhythmically either toward the body or away from the body (palm direction oriented accordingly). We further wanted to find out whether movement qualities and movement shape are related to the evaluative system in a similar way and with similar effect sizes. On the basis of Cacioppo et al. (1993) and Kestenberg-Amighi et al. (1999), we hypothesized a main effect for movement shape (approach vs. avoidance) and a main effect for movement quality (smooth vs. sharp rhythms): smooth rhythms, just like approach movements, were assumed to cause more positive attitudes.

## METHOD

### SAMPLE AND DESIGN

Forty participants (21 women, 19 men; mean age 22.90, SD = 7.37) were tested in a 2 × 2 design: independent variables were movement rhythms (smooth vs. sharp rhythms) and movement shape (approach vs. avoidance movement). Dependent variables were the offline-evaluation of the Chinese ideographs from the original experiment by Cacioppo et al. (1993; attitude measure), and an affect scale, including the seven original items related to movement qualities, and five new items related to movement shape (**Figure 2**). Participants, mostly students, had been recruited in the local pedestrian zone and in the psychology department and received either course credits or sweets as a reward.

### MOVEMENT MANIPULATION

In all four conditions participants were sitting, using both lower arms bilaterally (in parallel), which were moved in four (successive) steps rhythmically toward or away from the torso. Ten participants did an approach movement toward the body (palms also facing toward their body) combined with a smooth rhythm (round reversals, circular movement), 10 participants did an avoidance movement away from the body (palms facing away from their bodies) combined with the smooth rhythm. Ten participants performed an approach movement toward the body combined with a sharp rhythm (sharp reversals, angular movement), and 10 participants did an avoidance movement away from the body combined with a sharp rhythm.

### MATERIALS AND SCALES

#### Chinese ideographs

We employed the Chinese ideographs from the original study Cacioppo et al. (1993). This material had been tested in many – also international – contexts. We used 12 out of the 24 ideographs (the ones that had not been mirror imaged) and displayed them in a power point presentation at a rate of one ideograph every 10 s on a 2 m × 2 m screen at about 2 m distance from the observer. The entire duration of the presentation was 2 min. Departing from the original experiment, we did not use an initial evaluation during the first presentation of the ideographs.

#### Attitude scale

Participants had to rate the ideographs on a scale from 1 very negative to 6 very positive.

#### Affect scale

The affect scale used in the rhythms studies before was extended by five items related to change in movement shape (see **Figure 2**; Koch and Müller, 2007). The new items again were taken from the semantic interpretations of KMP-theory on the meaning of changes in movement shapes. Sample items were: *open vs. closed (offen vs. verschlossen), comfortable vs. uncomfortable (fühle mich wohl vs. fühle mich unwohl), inclined toward vs. disinclined (zugeneigt vs. abgeneigt*; see **Figure 2**; italicized items). *Cronbach’s Alpha* was 0.89.

### PROCEDURE

Participants met one of two experimenters (a man and a woman) and signed an informed consent sheet. Then they received the following information: “This is an experiment on the effects of arousal level on different dependent variables you are in the low arousal condition.” Subsequently, their pulse was taken (base rate) followed by a short training of the movement by one of the experimenters. Thereafter, they were told that they would now see a series of Chinese ideographs that they should merely watch and let them sink in. In a second circulation, they watched the ideographs while performing one of the four movements described above. Afterward, their pulse was taken again. Then they were asked to do the movement for a few more times, before they received the affect scale where they indicated their affect after the movement on the bipolar adjective scales. In a third circulation, they saw the ideographs again and had to rate them on a 6-point scale from very negative to very positive. On the final sheet, they provided their demographic data, received their reward, and were debriefed by one of the experimenters.

### DATA REDUCTION AND STATISTICAL ANALYSES

The sum scores of the affect items, and the means of the evaluations of the ideographs served as the basis for calculations. A MANOVA was computed with rhythm (smooth vs. sharp movement; sucking vs. biting) and shape (approach vs. avoidance movement; toward or away from one’s own body) as independent variables, and attitudes (evaluation of ideographs) and affect (MBAS) as dependent variables.

## RESULTS AND DISCUSSION

**FIGURE 4:**
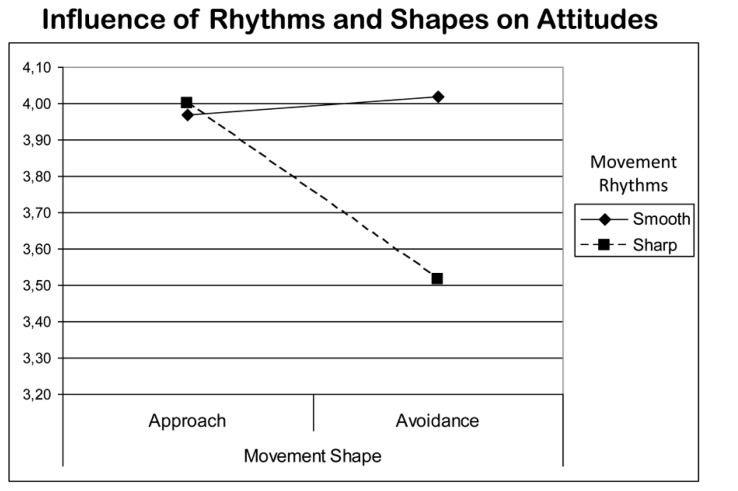
**Attitude ratings (*M*) in study 3: interaction of movement shape and movement quality (*N* = 40) for bilateral movement.** Ratings on a 6-point scale from 1 very negative to 6 very positive; *p < 0.05.

Results indicated that the movement condition had a systematic influence on attitudes and affect but not always in accordance with our expectations. While movement shape (approach vs. avoidance) had only a marginal influence on attitudes *F*(1,40) = 3.94; *p* = 0.055; η^2^ = 0.09, it did have a significant influence on the affect measure *F*(1,40) = 5.56; *p* = 0.024; η^2^ = 0.13: after the approach movement, participants felt significantly more relaxed, peaceful, etc.; after the avoidance movement, they felt significantly more tense, aggressive, etc. (no matter whether they had used indulgent or fighting rhythms). Movement rhythms unexpectedly had no influence on the affect measure (as we had seen in the two studies before), but significantly influenced the attitude measure: after using smooth rhythms, participants judged the initially valence-free ideographs more positively, than after using sharp rhythms *F*(1,40) = 4.63; *p* = 0.038; η^2^ = 0.11. The interaction of movement rhythms and shapes was significant for the attitude measure *F*(1,40) = 5.89; *p =* 0.020; η^2^ = 0.14 (see **Figure 4**).

The influence of rhythms on attitudes was a new finding. The influence of movement shape on affect is predicted by KMP-theory just the way it occurred in the experiment. An interesting finding is the interaction of movement rhythms and movement shape. It suggests that rhythm could be a moderator for shape in its effects on attitudes and potentially also on affect (**Table 2**). The effects were of comparable magnitude for movement quality and movement shape. In general, however, we only had a minimal sample size in study 3, leaving the power very low. Given the small effect sizes, results need replication.

**Table 2:** Descriptives of study 3 (*N* = 40).

	*Movement quality*		*Movement shape*	
	*Smooth rhythm*	*Sharp rhythm*	*Approach*	*Avoidance*
	*M (SD)*	*M (SD)*	*M (SD)*	*M (SD)*
Affect (sum)	24.76 (8.21)	27.20 (8.07)	23.00 (6.09)	28.96 (8.94)
Attitude (*M*)	3.99 (0.29)	3.73 (0.45)	3.96 (0.31)	3.74 (0.44)

*Affect (sum) = sum value of the affect scale; higher values indicate more negative affect; attitude (*M*) = mean evaluation of the 12 ideographs on 6-point scales. Movement shape had a significant influence on affect (*p* < 0.05); movement quality had a significant influence on attitude (*p* < 0.05); and the interaction of movement shape and movement quality was significant (*p* < 0.05).*

## GENERAL DISCUSSION

In this study, we investigated effects of dynamic body feedback, that is, effects from movement proper, on affect, attitudes and cognition. Based on KMP-theory, we introduced indulgent (smooth) and fighting (sharp) movement qualities as two basic principles from movement analysis (Kestenberg-Amighi et al., 1999). Movement qualities in general and movement rhythms with smooth vs. sharp reversals in particular were found to be important factors influencing affect and moderating effects of movement shape. Apart from the fact *which* movement is enacted (*shape*; here: approach vs. avoidance motor behavior), it seems equally important *how* the movement is enacted (*quality*). In sum, movement rhythms influenced the affect of participants and their attitudes toward initially valence-free stimuli, and moderated the influence of movement shape on attitude formation. Methodologically, the affect and attitude scale may be seen as measuring two aspects of a basic evaluation variable: affect as operationalized here can be seen as the self-related component of a dependent evaluative measure whereas attitude can be seen as the object-related component.

Studies 1 and 2 established smooth and sharp rhythms as basic dimensions of movement with differential effects on affect but not on cognition. It did not matter what particular pair of rhythms we selected or whether there was parallel or alternating limb action, smooth rhythms generally caused more positive (relaxed, peaceful, etc.) affect than sharp rhythms. Other operationalizations of cognitive variables may bear more potential to detect causal relationships between rhythms and cognition than the ones employed here. Following KMP-theory, fighting qualities could for example help people differentiate better by putting them in a more analytic mode and indulging qualities could lead people to blend categories more by putting them in a more integrative and intuitive mode. Overall, our findings are conform with KMP-theory, since Kestenberg (1995) assumes that movement rhythms are foremost associated with needs and affect.

Study 3 showed that body rhythms also influenced attitudes. However, in study 3, affect was not influenced by movement rhythms but by movement shape. Moreover, movement qualities and movement shape interacted significantly in their effects on attitudes. The magnitude of the influence of both independent variables seemed to be comparable in this first joined test. In the study of Cacioppo et al. (1993), participants provided an initial evaluation of the ideographs while watching them for the first time – a condition that the researchers established as necessary for the occurrence of the effect. Since the effects in our study occurred without the initial evaluation of the ideographs, it may be possible that dynamic movement manipulations have stronger effects than statically held postures. Given this is correct, it may be due to the greater naturalness of dynamic movement as part of our everyday experience: the missing spatio-temporal and kinesthetic features in held postures could be exactly the ones that are decisive for the occurrence of the effect. However, because of the merely marginal significance of movement shape on attitudes (*p* = 0.055), and the small sample size, a replication of the same study with a larger sample is needed in order to analyze the complex influences of movement qualities and movement shape on attitudes and affect. As a next step, it may be useful to separate rhythms and shape manipulations within one design to find out more about potential hierarchies among the effects.

### BEYOND STATIC BODY FEEDBACK

On a theoretical level, our results underline the importance of effects from movement shape (e.g., Neumann and Strack, 2000; Raab and Green, 2005; Friedman and Elliot, 2007) and complement the picture by adding movement qualities to the tradition of embodiment research. Movement qualities clearly modify the meaning of movements adding a second semantic dimension (cf. Suitner et al., 2012). Our studies are among the first to demonstrate an influence of dynamic movement quality in a body feedback context and the first using a differentiated theory background on how movement maps to semantics to derive its’ predictions. The theory employed in this research and our empirical findings are compatible with other recent theoretical approaches in psychology and the neurosciences (e.g., Damasio, 1994; Barsalou, 1999; Gallese and Lakoff, 2005). All of these approaches assume action and action simulation in sensory-motor areas of the brain at the basis of affect, thinking and reasoning, while the mere duplication of information in abstract symbols, as postulated by amodal theories, is assumed to be implausible and uneconomic.

### CLINICAL APPLICATIONS

Approach and avoidance motor behavior in interaction with movement qualities can take on different meaning in clinical contexts and psychopathology. To a borderline patient approach movements with sharp rhythm may cause more positive affect when he/she is in a state where self-harm is a goal or a means of relief. The obsessive-compulsive patient may benefit from smooth avoidance movements in order to overcome compulsive approach actions. In general, movement qualities employed in therapy can be assumed to cause changes in affect and attitudes. This assumption and its long term implications need to be explored in future clinical studies.

Approach and avoidance movements have self-related and interpersonal affective implications. Self-related implications have been described above, interactional implications have been described for example by Kafka (1950). Kafka dealt with the basic affects (*Uraffekte*) and assumed four of them, two approach- and two avoidance-related ones:

• *profusion*: “along with me to you” (love, affection);
• *ingestion*: “along with you to me” (desire, greed);
• *recession*: “away with me from you” (fear, disgust);
• *ejection*: “away with you from me” (anger, hatred).

These tendencies are picked up in the works of Shai and Belsky (2011), and Fuchs and Koch (2014; this issue) on embodied affectivity, both emphasizing the huge overlap between affect and motor action in general (see also Cipolletta, 2013), and the influence of intersubjective factors in particular. Such intersubjective factors are presently investigated in our research on movement rhythm and their communicative functions in handshakes and embraces (Koch, unpublished).

In psychopathology, patients often get stuck in one self-related or interpersonal way of being. One clinical goal would be to have chronically stuck patients expand their movement repertoire in order to extend their action and affective options, coping mechanisms, self-efficacy, and sense of agency. In dance movement therapy, changes in movement are assumed to produce global and specific changes in affect, attitudes and cognition, as differentially predicted by the KMP (Kestenberg, 1995) or Laban Movement Analysis (Laban, 1960); many of these assumptions still need empirical testing.

### CONCLUSION

This research investigated the influence of movement on affect, attitudes and cognition, extending previous, more statically focused work on the effects of motor behavior. It extends Aronoff et al.’s (1992) and other researchers’ findings on the basic smooth/sharp distinction with a body feedback approach. The general aim of the studies was to investigate the meaning of movement qualities, here in particular whether movement rhythms with smooth vs. sharp reversals are basic dimensions of movement with differential implications for affect, attitudes and cognition (as also evidenced from other lines of empirical studies such as Köhler, 1929; Aronoff et al., 1992; Bar and Neta, 2006). Merleau-Ponty (1965) assumed that each experience of a quality is in reality an experience of a certain way of movement. Is it possible that we have greatly overlooked the meaning of movement in clinical psychology? Could this be due to the fact that the dynamics of body movement cannot easily be investigated with classic experimental methods but in fact would be more appropriately modeled within a dynamic systems theory framework and methods? Our findings trace effects that start as movement rhythms in the body: in the alternation of muscle tension and relaxation and its smooth vs. sharp reversals dependent on our organisms’ need to indulge or to separate. The results from our findings indicate that dynamic movement and movement qualities are an important research topic with potentially far reaching implications for clinical and health-related questions, but also for social cognition, interaction, *communication*, thinking, learning, memory, research methods, and in any case – as demonstrated here – for affect and attitudes as core themes of social embodiment research.

## Conflict of Interest Statement

The author declares that the research was conducted in the absence of any commercial or financial relationships that could be construed as a potential conflict of interest.

## Footnotes

1 Most movement analysis systems have been developed in dance-related contexts. Just like note writing in music, there was a need for notational systems of movement in dance; movement analysis developed alongside movement notation (Laban, 1960; ).

2 The 10 rhythms corresponding to 10 developmental movement stages are: sucking, snapping/biting, twisting, straining-release, running–drifting, starting–stopping, swaying, surging/birthing, jumping and spurting/ramming (Kestenberg and Sossin, 1973).

3 The word *congruent* is always used in the sense that the content of the answers matches the assumed semantics of the movement.
